# Accuracy of Systemic Inflammatory Indices in identifying Neonatal Sepsis in preterms in a tertiary care hospital in Mangalore

**DOI:** 10.12688/f1000research.162331.1

**Published:** 2025-04-03

**Authors:** Smrithi GM, Gayathri Renganathan, Smitha D'sa

**Affiliations:** 1 Kasturba Medical College Mangalore, Manipal Academy of Higher Education, Manipal, India; 2 Department of Pediatrics, Kasturba Medical College Mangalore, Manipal Academy of Higher Education, Manipal, India

**Keywords:** Neonatal Sepsis, Prevention, Preterm, Inflammatory markers, Early diagnosis

## Abstract

**Background:**

Both term and preterm neonates can develop sepsis, a potentially lethal and life-threatening condition, during the first 28 days of life. Neonatal sepsis accounts for 8% of all neonatal fatalities. This study aimed to evaluate the predictive power of lab-based diagnostic indices for neonatal sepsis in preterm infants.

**Methods:**

The Systemic Inflammatory Indices of the two groups of preterms – one control group without sepsis and one case group with sepsis–were compared to assess their value in predicting Neonatal Sepsis. Data from 138 preterm neonates were used in the present study. Systemic Inflammatory Indices were calculated and compared from the collected data in both the case and control groups.

**Results:**

Platelet count, Pan Immune Inflammation Value (PIV), Platelet to Lymphocyte Ratio (PLR) and Systemic Immune Inflammatory Index (SII) were found to be significant predictors of neonatal sepsis. Platelet count had the highest predictive value, with an AUC value of 0.715 and optimal cut-off value of 219500. It had a sensitivity of 75.4 and specificity of 65.2.

**Conclusion:**

According to this study, sepsis in preterm infants can be predicted by using systemic inflammatory indices. This will aid in early sepsis diagnosis and management and, in turn, reduce neonatal morbidity and mortality associated with sepsis.

## Introduction

Dysregulation of the host response to any systemic bacterial, viral, or fungal infection within day 28 of the life of both term and preterm newborns can result in neonatal sepsis, a potentially fatal and life-threatening illness. ^1^ Neonatal sepsis is classified into two groups depending on when it manifests after delivery: early onset sepsis (EOS) and late-onset sepsis (LOS). EOS is the term for neonatal sepsis diagnosed at or before 72 hours of life (some experts have defined it as 7 days), whereas LOS is the term for sepsis that is diagnosed at or after 72 hours of life. ^2^

Neonatal sepsis accounts for approximately 8% of all neonatal fatalities. Particularly in low- and middle-income nations, it is the primary cause of neonatal morbidity and mortality. ^3^ The approximate statistic for EOS is 2,496 in 100,000 live births, that is 2.6 times greater than the incidence of LOS, which is 946 per 100 live births, per a systematic review & meta-analysis. ^4^ The highest rate of clinical sepsis (17,000per 1,000,000 live births) has been reported in India. ^5^ The fatality rate due to sepsis in Indian newborns ranges from 25% to 65%. ^6^

The gold standard for diagnosing neonatal sepsis is to obtain a positive blood culture. ^7^ The normal turnaround time for blood culture findings is 48–72 h after collection, and the lack of distinct clinical symptoms makes early diagnosis of sepsis difficult. ^8, 9^ Therefore, a sensitive and simple bedside diagnostic tool is required for early detection of newborn sepsis.

According to the analysis by Zhu et al., newborn sepsis may be reliably predicted by the neutrophil-to-lymphocyte ratio (NLR), Systemic Immune Inflammatory Index (SII), and platelet-to-lymphocyte ratio (PLR). ^10^ Pan immune Inflammation Value (PIV), Monocyte to Lymphocyte Ratio (MLR), and Systemic Inflammation Response Index (SIRI) were also found to be higher in the early onset sepsis (EOS) group of neonates in a study by Cakir et al. ^11^ We aimed to determine whether the SII, SIRI,MLR, NLR, PIV, and PLR are valuable markers for the early diagnosis of neonatal sepsis in preterms in the Indian population.

### Summary of evidence

The investigation conducted by Zhu et al. suggested that the platelet-to-lymphocyte ratio (PLR), Systemic Immune Inflammatory Index (SII), and neutrophil-to-lymphocyte ratio (NLR) can all be used to accurately predict newborn sepsis; the highest predictive value was the SII. ^10^ An accessible and credible systemic inflammatory index for the diagnosis of EOS in Very Low Birth Weight preterm newborns is SIRI, when combined with other indicators, as per a study by Cakir et al. ^11^ A study by Aydogan et al. found that NLR and SII have predictive power for identifying neonatal sepsis in infants with CHD. ^12^

Güngör et al. established that the SII can be used to predict UTIs in babies. ^13^ A research by Runqiang Liang et al. found that SII is crucial for diagnosing serious bacterial infections in newborns. ^14^ Cakir et al. found that a higher SII level (≥78.2) may indicate the development of RDS in preterm newborns. ^15^ It was found that SII, SIRI, PIV, and NLR were significantly increased in infants with hypoxic-ischemic encephalopathy when compared to the control group in a study by Burak Ceran et al. ^16^ In a study conducted among 2164 premature infants, Chen et al. reported that elevated SIRI and SII values were correlated with an increased risk of secondary infections. ^17^

A literature review by Muzaffer Islam et al. concluded that SII, NLR, SIRI, and PLR are useful for predicting outcomes in inflammation-related conditions. ^18^ Tanacan et al. observed that SII could be a potential biomarker for predicting unfavorable neonatal outcomes in PPROM. ^19^ The NLR, SII, and PLR were revealed to be independent indicators of sepsis-related mortality by Mangalesh et al. SII also has an incremental effect on the Sequential Organ Failure Assessment (SOFA) score in their study. ^20^ In their research, Xianghui Liang et al came to the conclusion that WBC and platelet counts on day one of sepsis are useful markers for predicting fatality in neonates with sepsis. ^21^ Cruz et al. found that no total count parameters at commonly used thresholds identified infants with Invasive Bacterial Infection (IBIs) with sufficient accuracy. ^22^ ANC, thrombocytes, and TLC exhibited good diagnostic sensitivity and specificity for newborn sepsis according to a study by Minichil Worku et al. ^23^ Vizcarra-Jimenez et al. found that thrombocytopenia was associated with higher mortality rates in neonates with sepsis. ^24^ In newborn sepsis, thrombocytopenia quadruples the chance of mortality, and in gram-negative sepsis, the risk of mortality increases by six times, as reported by Isabelle M C Ree et al. ^25^ NLR and PLR showed substantial diagnostic utility in a study conducted by Ashour et al. ^26^ Can et al. discovered in their study that NLR and PLR were positively associated with early onset sepsis. ^27^ In a clinical investigation of premature infants, Vardar et al. revealed that the SII values of the late-onset sepsis (LOS) group were significantly higher than those in the control group. ^28^ Neonatal sepsis was strongly associated with leukopenia, thrombocytopenia, anemia, and a high Monocyte Distribution Width (MDW) score, based on a study by Mubaraki et al. ^29^ Elevated NLR levels are associated with an increased risk of newborn sepsis according to data gathered by Li et al. ^30^

### Need for study

No recent study has demonstrated the utility of Systemic Inflammatory Markers (SIMs) as reliable predictors of Neonatal Sepsis in the Indian population. This study will help determine whether SIMs can be used as an early sensitive predictor of sepsis in preterms and will be useful in predicting neonatal sepsis at the bedside. This will also help reduce neonatal morbidity and mortality.

## Methods

The Neonatal Intensive Care Unit (NICU), Government Lady Goschen Hospital, Mangalore, was the site of this case–control study. The case group consisted of neonatal sepsis preterm infants, and the control group consisted of preterm infants without sepsis. The study duration was six months. The sample size included in the study consisted of 138 preterm neonates, 69 preterm neonates with sepsis, and 69 preterm neonates without sepsis. Open Epi Version 3.01 was used to calculate the sample size. The sample size was calculated three times using three different variables, out of which the highest sample size was chosen. ^10^ The inclusion criteria for selection were preterm neonates admitted to NICU. Term neonates admitted to the NICU, healthy newborns in postnatal wards, and those unwilling to participate in the study were excluded from the study.

Infants born preterm are born before 37 weeks of gestation. They were further categorized as extremely preterm (<28 weeks), very preterm (28-31 weeks), late (34-36 weeks), and moderately preterm (32-33 weeks). ^31^ The “gold standard” to confirm neonatal sepsis is still the conventional culture methods. If microbial growth is observed in blood cultures or other sterile body fluids, sepsis is considered culture-proven. ^32^

Preterms will be divided into a case group consisting of 69 preterms with sepsis and a control group consisting of 69 preterms without sepsis based on their blood culture reports sent according to the NICU protocol. Preterms with positive blood cultures will be in the case group, and preterms with negative blood culture reports and no other clinical signs of sepsis will be selected for the control group. ^33^

Samples will be collected from the neonate after 24 h of life as per the routine newborn screening protocol. Data were obtained from neonatal health records. From the collected data, the SII, SIRI, PIV, NLR, MLR, and PLR were calculated using the following formulae:

1)
$\text{Systemic Immune Inflammatory Index}\;\left(\mathrm{SII}\right)={\left[\text{platelet}/\text{lymphocyte}\right]}^{*}\;\text{Neutrophil}$ ^34^
2)
$\text{Systemic Inflammation Response Index}\;\left(\text{SIRI}\right)={\left[\text{monocyte}/\text{lymphocyte}\right]}^{*}\;\text{Neutrophil}$ ^35^
3)
$\mathrm{Pan}\;\text{Immune Inflammation Value}\;\left(\mathrm{PIV}\right)={\text{Platelet}}^{*}\;{\left[\text{monocyte}/\text{lymphocyte}\right]}^{*}\;\text{Neutrophil}$ ^36^
4)
Neutrophil to Lymphocyte Ratio(NLR) ^10^
5)
Platelet to Lymphocyte Ratio(PLR) ^10^
6)
Monocyte to Lymphocyte Ratio(MLR) ^11^

IBM SPSS (Statistical Package for Social Sciences) Statistics for Windows Version 29.0. Armonk, NY:IBM Corp. used to analyse the data. Descriptive statistics were presented as standard deviations and means. An independent sample t-test was used to compare the scores of the case and control arms. Statistical significance was defined as a p-value of less than 0.05. To assess the predictive ability of the diagnostic markers, ROC (Receiver Operating Characteristic) analysis was performed. Additionally, the optimum cut-off value was determined using the Youden Index.

## Results

A total of 138 preterm neonates were included in this study, out of which, 50% of patients belonged to the sepsis group.

Table 1 shows the clinical characteristics of the neonates. Of the 69 infants in the control group, 49.3% were male. The babies were categorized as late preterm (52.2%), moderate preterm (18.8%), very preterm (27.5%), and extremely preterm (1.4%). The majority of babies were delivered via normal vaginal delivery (60.9%). There were 38 babies with low birth weight (LBW), 29 with very low birth weight (VLBW), one with extremely low birth weight (ELBW) baby and 1 baby of normal birth weight. 31.9 Of the babies, 31.9% were found to be small for gestational age (SGA) and 68.1% were found to be appropriate for gestational age (AGA).

**Table 1.** Clinical characteristics of the neonates included in the study. ^37^

	Sepsis n = 69 n (%)	Control n = 69n (%)
Mother’s age (Mean (SD))	26.46 (5.41)	28.06 (4.86)
Gender of neonate		
Male	35 (50.7)	34 (49.3)
Female	34 (49.3)	35 (50.7)
Preterm category		
Late preterm (34-37 weeks)	23 (33.3)	36 (52.2)
Moderate preterm (32-33 weeks)	16 (23.2)	13 (18.8)
Very preterm (28-31 weeks)	29 (42)	19 (27.5)
Extremely preterm (<28 weeks)	1 (1.4)	1 (1.4)
Mode of delivery		
Normal	39 (56.5)	42 (60.9)
LSCS	30 (43.5)	27 (39.1)
Birth weight		
LBW	14 (20.3)	38 (55.1)
VLBW	54 (78.3)	29 (42)
ELBW	1 (1.4)	1 (1.4)
NBW	0 (0)	1 (1.4)
SGA/AGA/LGA		
SGA	14 (20.3)	22 (31.9)
AGA	55 (79.7)	47 (68.1)

50.7 Of the patients in the sepsis group, 50.7% were male. Preterm sepsis was categorized as late preterm (33.3%), moderate preterm (23.3%), very preterm (42%), and extremely preterm (1.4%). None of the infants with sepsis had a normal birth weight. The majority of babies were delivered via normal vaginal delivery (56.5%). There were 14 LBW infants, 54 VLBW infants, and 1 ELBW infant. 20.3 Of the infants, 20.3% were small for gestational age (SGA) and 79.7% were appropriate for gestational age (AGA).

Table 2 shows laboratory parameters of the neonates. Compared to the control group, preterm babies of sepsis group had lower WBC, lower monocyte, lower neutrophil, lower platelet and lymphocyte counts. Values of platelet counts (P < 0.001), SII (P = 0.002), PIV (P < 0.001) and PLR (P < 0.001) were found to be statistically significant. Values of total count (P = 0.116), monocyte (P = 0.197), neutrophil (P = 0.692), lymphocyte (P = 0.434), SIRI (P = 0.942), NLR (P = 0.58) and MLR (P = 0.871) were not statistically significant.

**Table 2.** Laboratory parameters. ^37^

	Control (Median (IQR))	Case (Median (IQR))	P value
TOTAL COUNT	11500 (8275-16390)	9670 (6600-16710)	0.116
MONOCYTE	8 (5.5-10)	6 (4.5-10)	0.197
NEUTROPHIL	59 (49.5-66.5)	56 (45-70)	0.692
PLATELET	248000 (197500-303000)	164000 (66500-229000)	<0.001
LYMPHOCYTE	28 (21-36)	25 (17-36.5)	0.434
SII (SYSTEMIC IMMUNE INFLAMMATORY INDEX)	92233.7 (92233.7-92233.7)	92233.7 (92233.7-92233.7)	0.002
SIRI (SYSTEMIC INFLAMMATION RESPONSE INDEX)	16 (8.9-25.6)	14.9 (4.9-32.4)	0.942
PIV (PAN IMMUNE INFLAMMATION VALUE)	9223.4 (9223.4-9223.4)	9223.4 (9223.4-9223.4)	<0.001
NLR (NEUTROPHIL TO LYMPHOCYTE RATIO)	2.1 (1.4-3.2)	2.2 (1.2-3.9)	0.58
PLR (PLATELET TO LYMPHOCYTE RATIO)	8833.3 (4809-9223.4)	4939.4 (2577.7-9223.4)	<0.001
MLR (MONOCYTE TO LYMPHOCYTE RATIO)	0.3 (0.2-0.4)	0.3 (0.1-0.5)	0.871

To evaluate the predictive power of SII, platelet count, PIV, and PLR for neonatal sepsis, the area under the curve (AUC) values were calculated from the receiver operating characteristic (ROC) curves. The cutoff points were determined using the Youden Index.

**Figure 1.**
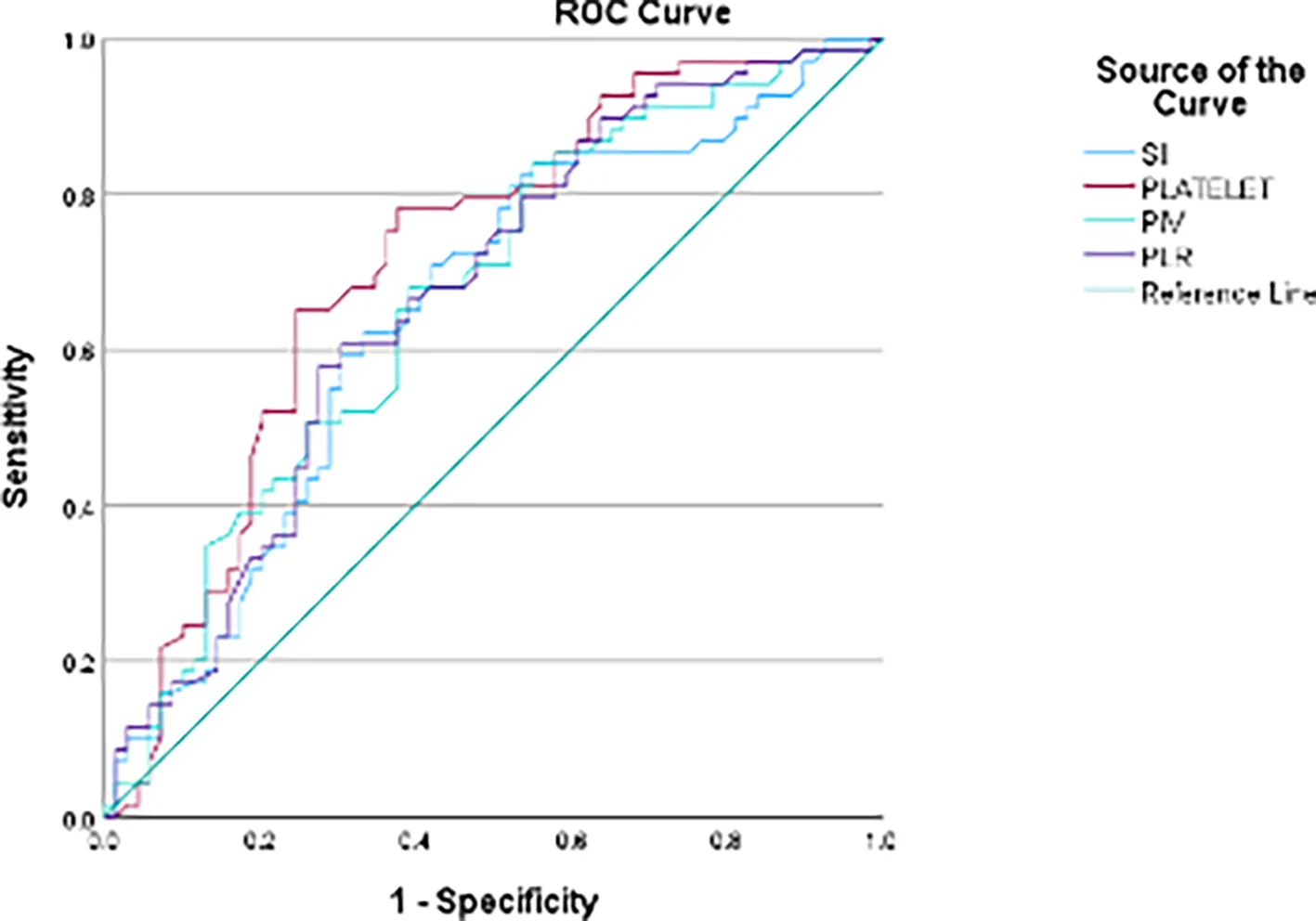
ROC curve analysis. ^37^

The ROC curves for the predictive ability of SII, platelet count, PIV, and PLR are shown in Figure 1. Tables 3 and 4 show the ROC analysis and optimal cutoff values. Of the four variables, platelet count had the highest AUC value (0.715), with an ideal cut-off concentrations of 219500, and sensitivity and specificity of 75.4 and 65.2 respectively. The AUC value for PLR was 0.668, the sensitivity and specificity were 72.5 and 58%, respectively, and the ideal cut-off value was 7923.19. The AUC value and cut-off value for PIV were 0.665 and 2187333.33, respectively, with a sensitivity of 60.9 and specificity of 68.1. The AUC value was lowest for SII (0.65), with an ideal cut-off values of 37656.79, and sensitivity and specificity of 66.7 and 62.3%, respectively

**Table 3.** ROC analysis to assess predictive ability of diagnostic markers to predict neonatal sepsis. ^37^

Parameter	AUC	p value	95% CI	
			Lower bound	Upper bound
SII	0.65	0.002	0.558	0.743
Platelet	0.715	<0.001	0.628	0.802
PIV	0.665	0.001	0.574	0.755
PLR	0.668	0.001	0.577	0.758

**Table 4.** Optimal cutoff determined using ROC analysis. ^37^

Parameter	Cut-off	Sensitivity	Specificity
SII	37656.79	66.7	62.3
Platelet	219500	75.4	65.2
PIV	2187333.33	60.9	68.1
PLR	7923.19	72.5	58.0

### Critical reflection

Of the 138 preterm babies included in the study, the majority were late preterm, 23 belonged to the sepsis group, and 36 belonged to the control group. Most of the babies in both the sepsis (56.5%) and control (60.9%) groups were delivered by normal vaginal delivery. The majority of babies in the sepsis group had very low birth weight (78.3%) and the majority in the control group had low birth weight (55.1%).

Platelet count, SII, PIV, and PLR were found to be significant predictors of neonatal sepsis. Platelet count had the highest predictive value, with an AUC value of 0.715 and optimal cut-off value of 219500. It had a sensitivity of 75.4 and specificity of 65.2.

## Discussion

Given that blood cultures take time to produce findings and sepsis symptoms are not very specific, research has been conducted to identify an easier bedside test that can accurately detect neonatal sepsis. This study showed that the platelet count, PLR, PIV, and SII can be used to predict neonatal sepsis.

Our study found that platelet counts had a greater predictive ability for neonatal sepsis than PLR, PIV, and SII. This was in line with the findings of Liang et al. and Worku et al., who also concluded that platelet count was a good marker for the diagnosis and prognosis of neonatal sepsis. ^21, 23^ Vizcarra-Jimenez et al. and Isabelle M C Ree et al. found that thrombocytopenia was a significant predictive factor for newborn sepsis. Our study had also found that AUC values were highest for platelet counts. ^24, 25^

Zhu et al. claimed that the SII had the highest predictive value for neonatal sepsis and that sepsis may be reliably predicted using NLR and PLR. ^10^ According to our findings, platelet count had the highest predictive value. The PLR and SII also showed statistically significant differences. However, NLR levels were not significant in our findings. According to Liang et al., the SII is essential for identifying infections in infants. ^14^ Our research found similar results for SII. Additionally, our research revealed that while SII was a strong predictor of infant sepsis, platelet count was a more important indicator.

Chen et al. discovered that high SIRI and SII values were associated with an increased risk of infections in preterms, but our study found that only SII was a significant sepsis predictor and not SIRI. ^17^ In a study involving premature babies conducted by Vardar G et al., SII values were more crucial in the sepsis group than in the control group. ^28^ Similar findings on the SII were found in our study.

SII, PLR, and NLR were independent predictors of newborn sepsis, as reported by Islam et al. and Mangalesh et al. ^18, 20^ Our investigation did not demonstrate that the NLR was an independent predictor, even though the SII and PLR values were significant and comparable to those of their studies. Ashour et al. and Can et al. revealed that NLR and PLR are positively associated with sepsis. ^26, 27^ The results of our ROC curve analysis were comparable for PLR but not for NLR. NLR was found to be an important index for predicting sepsis according to Li et al. ^30^ However, based on our analysis, NLR values were not significant in our study population.

Platelet counts have proven to be helpful in the early detection of neonatal sepsis in our setup, which is a tertiary care hospital located in a coastal area. This demonstrates the significance and increased necessity of utilizing thrombocyte count and thrombocyte-related variables for early neonatal screening in the NICU.

### Limitations

This study was performed only among preterm infants in the NICU, and studies involving term infants admitted to the NICU may be required. This study was conducted at a public institution in a coastal area. Further multicenter studies with larger patient populations are warranted.

## Conclusion

The results of this study show how platelet counts, PLR, PIV, and SII can be used to predict sepsis in newborns. The early detection and treatment of newborn sepsis may be helpful markers. According to this study, sepsis in preterm infants can be predicted using systemic inflammatory indices. This will aid in early sepsis diagnosis and management and, in turn, reduce neonatal morbidity and mortality associated with sepsis.

## Ethical approval statement

On 17/10/24, ethical clearance was granted by The Institutional Ethics Committee at Kasturba Medical College in Mangalore (Protocol No: IECKMCMLR10/2024/606). The MS of the Government Lady Goschen Hospital has given us permission to conduct this study.

## Consent statement

Consent is not required for this study as it is a lab report based study and no intervention was done. The Institutional Ethics Committee at Kasturba Medical College Mangalore has approved this study.

## Data Availability

Figshare: Data – Excel Sheet (Neonatal sepsis research data Excel) https://doi.org/10.6084/m9.figshare.28395161.v1. ^37^ The project contains the following underlying data:
• Sepsis Data 2 Data are available under the terms of the Creative Commons Attribution 4.0 International license (CC-BY 4.0).
