# Accuracy of Systemic Inflammatory Indices in identifying Neonatal Sepsis in preterms in a tertiary care hospital in Mangalore

**DOI:** 10.12688/f1000research.162331.3

**Published:** 2026-06-10

**Authors:** Smrithi GM, Gayathri Renganathan, Smitha D'sa

**Affiliations:** 1 Kasturba Medical College Mangalore, Manipal Academy of Higher Education, Manipal, India; 2 Department of Pediatrics, Kasturba Medical College Mangalore, Manipal Academy of Higher Education, Manipal, India

**Keywords:** Neonatal Sepsis, Prevention, Preterm, Inflammatory markers, Early diagnosis

## Abstract

**Background:**

The utility of Systemic Inflammatory Indices as accurate indicators of neonatal sepsis remains underexplored in the Indian population. This study will assist in determining whether these indices can be used to predict neonatal sepsis at the bedside and as an early sensitive predictor of sepsis in preterms.

**Methods:**

A prospective case-control study was done, where the Systemic Inflammatory Indices of the two groups of preterms – one control group without sepsis and one case group with culture proven sepsis–were compared to assess their value in predicting Neonatal Sepsis. Data from 138 preterm neonates were used in the present study. Six Systemic Inflammatory Indices were calculated - Systemic Immune Inflammatory Index (SII), Systemic Inflammation Response Index (SIRI), PanImmune Inflammation Value (PIV), Neutrophil to Lymphocyte Ratio (NLR), Platelet to Lymphocyte Ratio (PLR) and Monocyte to Lymphocyte Ratio (MLR). These values from both the case and control groups were compared.

**Results:**

Platelet count had the highest predictive value, with an AUC value of 0.715(95% CI 0.628-0.802, p<0.001) and optimal cut-off value of 219500 cells/cu mm. It had a sensitivity of 75.4 and specificity of 65.2. PIV had an AUC of 0.665(95% CI 0.574-0.755, p=0.001), a sensitivity of 60.9, and a specificity of 68.1. For PLR, Sensitivity and specificity were 72.5 and 58, respectively, with an AUC of 0.668(95% CI 0.577-0.758, p=0.001). With sensitivity and specificity of 66.7 and 62.3 respectively, SII had an AUC of 0.65(95% CI 0.558-0.743, p=0.002). SIRI, NLR and MLR did not demonstrate statistically significant differences between the groups(p>0.05).

**Conclusion:**

There was moderate correlation between the studied hematological indices and positive cultures, suggesting their potential role as inflammatory markers. Larger prospective trials should be conducted to further validate their potential clinical value.

## Introduction

Dysregulation of the host response to any systemic bacterial, viral, or fungal infection within day 28 of the life of both term and preterm newborns can result in neonatal sepsis, a potentially fatal and life-threatening illness. ^1^ It is categorized based on onset - early-onset sepsis (EOS), diagnosed at or before 72 hours of life (some define as 7 days), and late-onset sepsis (LOS), diagnosed after 72 hours. ^2^

Neonatal sepsis accounts for approximately 8% of neonatal deaths and remains a major cause of neonatal morbidity and mortality, especially in low- and middle-income countries, with early-onset sepsis occurring more frequently than late-onset sepsis. ^3, 4^

The highest rate of clinical sepsis (17,000per 1,000,000 live births) has been reported in India. ^5^ The fatality rate due to sepsis in Indian newborns ranges from 25% to 65%. ^6^

Blood culture remains the gold standard for diagnosis, ^7^ but results take 48–72 hours, and lack of distinct early clinical signs make timely diagnosis difficult. ^8, 9^ Therefore, a sensitive and simple bedside diagnostic tool is required for early detection of newborn sepsis.

Studies show that neonatal sepsis, especially early-onset sepsis (EOS), can be predicted using inflammatory markers such as NLR, PLR, SII, MLR, SIRI, and PIV, which are typically elevated. ^10, 11^ We aimed to determine whether the SII, SIRI, MLR, NLR, PIV, and PLR can be used as valuable markers for the early diagnosis of neonatal sepsis in preterms in the Indian population.

### Summary of evidence

The prognostic significance of NLR and SII for newborn sepsis in babies with congenital heart disease was shown by Aydogan et al. ^12^ Güngör et al. found SII to be a predictor of urinary tract infections in neonates, ^13^ while Runqiang Liang et al. highlighted its role in diagnosing serious bacterial infections. ^14^ In premature infants, elevated SII levels were also linked to respiratory distress syndrome. ^15^ Increased SII, SIRI, PIV, and NLR values were observed in infants with hypoxic–ischemic encephalopathy. ^16^ Higher SIRI and SII levels were linked to a higher risk of secondary infections in preterm newborns, according to Chen et al. ^17^

SII, NLR, SIRI, and PLR were validated by Muzaffer Islam et al., and SII was linked to adverse neonatal outcomes in PPROM by Tanacan et al. ^18, 19^ Mangalesh et al. identified NLR, SII, and PLR as mortality predictors, while Xianghui Liang et al. noted day-1 WBC and platelets as neonatal sepsis mortality markers. ^20, 21^ Cruz et al. found leukocyte counts alone were not reliable for infection, while Minichil Worku et al. found TLC, ANC, and platelets useful for neonatal sepsis diagnosis. ^22, 23^ Thrombocytopenia was consistently associated with increased mortality in neonatal sepsis, particularly in gram-negative infections, as reported by Isabelle M C Ree et al and Vizcarra-Jimenez et al. ^24, 25^ Ashour et al and Can et al found that NLR and PLR showed significant diagnostic utility and were positively associated with early-onset sepsis. ^26, 27^ Vardar et al revealed that preterm neonates with late-onset sepsis were shown to have higher SII levels. ^28^ Mubaraki et al. reported strong associations between neonatal sepsis and leukopenia, thrombocytopenia, and anemia, while Li et al. found a significant association with increased NLR. ^29, 30^

### Need for study

Due to paucity of data in the Indian context, the utility of Systemic Inflammatory Indices as reliable predictors of Neonatal Sepsis in preterm infants has not been adequately evaluated this study will help determine whether these indices can be used as an early sensitive predictor of sepsis in preterms and will be useful in predicting neonatal sepsis at the bedside. This will also help reduce neonatal morbidity and mortality.

## Methods

The Neonatal Intensive Care Unit (NICU), Government Lady Goschen Hospital, Mangalore, was the site of this prospective case–control study. The case group consisted of neonatal sepsis preterm infants, and the control group consisted of preterm infants without sepsis. The study duration was six months. The sample size was calculated using OpenEpi version 3.01. Effect estimates for inflammatory indices were derived from a previously published study by Zhu et al., ^10^ which evaluated NLR, PLR, and SII as diagnostic markers of neonatal sepsis. Sample size calculations were performed separately for three inflammatory indices, and the largest calculated sample size was selected to ensure adequate statistical power. Based on this approach, a total of 138 preterm neonates (69 cases and 69 controls) were included in the study.

The inclusion criteria for selection were preterm neonates admitted to NICU. Term neonates admitted to the NICU, preterm neonates with incomplete laboratory records and those in whom blood culture was not performed, neonates with major congenital anomalies, healthy newborns in postnatal wards, and those unwilling to participate in the study were excluded from the study.

Infants born preterm are born before 37 weeks of gestation. They were further categorized as extremely preterm (<28 weeks), very preterm (28-31 weeks), late (34-36 weeks), and moderately preterm (32-33 weeks). ^31^ The “gold standard” to confirm neonatal sepsis is still the conventional culture methods. If microbial growth is observed in blood cultures or other sterile body fluids, sepsis is considered culture-proven. ^32^

**Figure 1.**
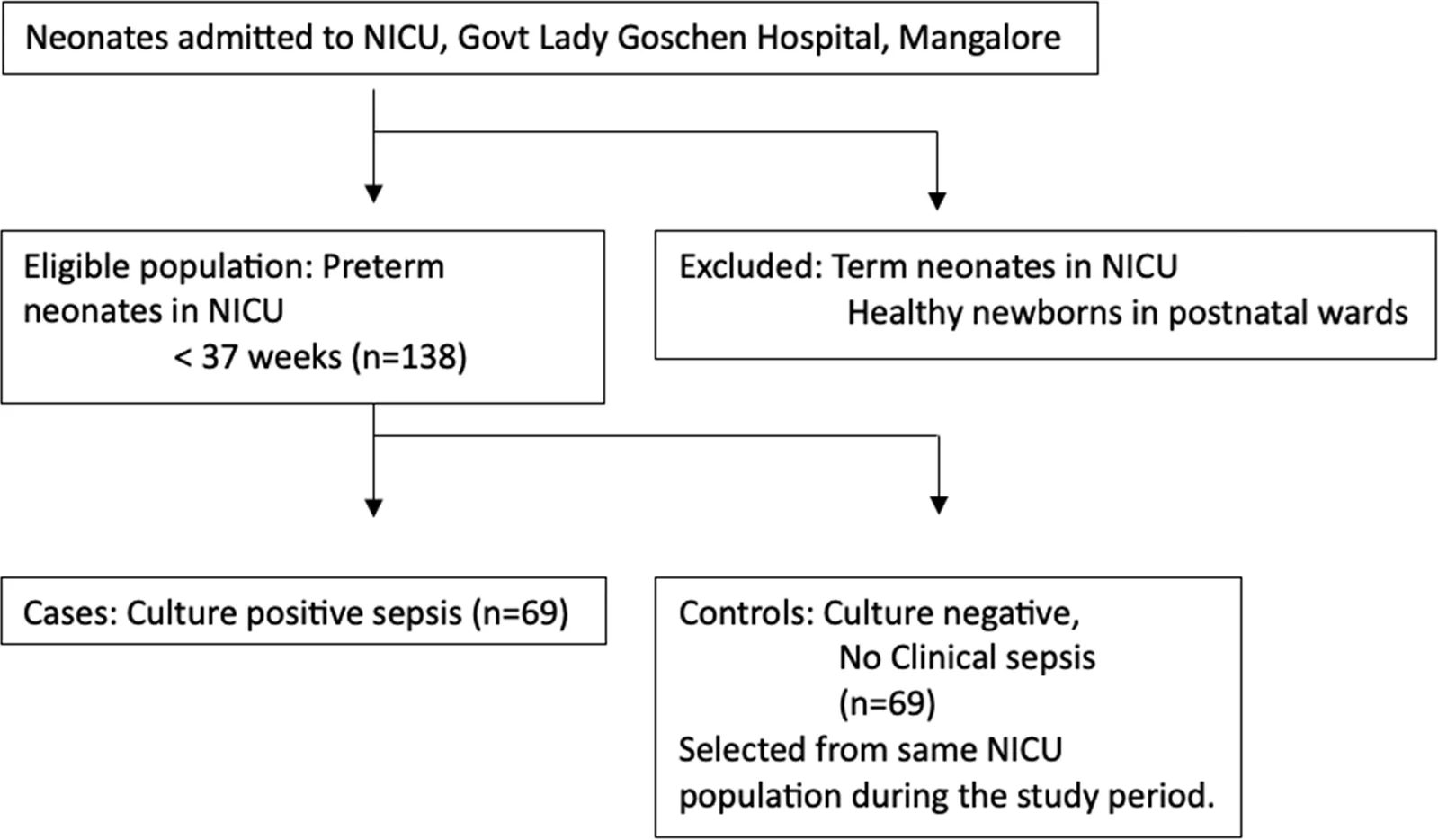
Flow diagram showing selection of cases and controls among preterm neonates admitted to the NICU.

Preterms were divided into a case group consisting of 69 preterms with sepsis and a control group consisting of 69 preterms without sepsis based on their blood culture reports sent according to the NICU protocol. Preterms with positive blood cultures were in the case group, and preterms with negative blood culture reports and no other clinical signs of sepsis were selected for the control group ( Figure 1). ^33^ Formal matching was not performed; however, both cases and controls were drawn from the same source population of preterm neonates admitted to the NICU during the same study period.

Samples were collected from the neonate after 24 h of life as per the routine newborn screening protocol, prior to confirmation of sepsis by blood culture at the Government Lady Goschen Hospital NICU, blood culture is performed as part of routine neonate screening protocol, consistent with recommendations for high risk preterm monitoring in resource constrained settings. Data were obtained from neonatal health records. These values reflect the inflammatory milieu at the time of clinical suspicion and blood culture collection, rather than post confirmation. Blood cultures were performed using the BACTEC system following NICU protocol. From the collected data, the SII, SIRI, PIV, NLR, MLR, and PLR were calculated using the following formulae:

1)
$\text{Systemic Immune Inflammatory Index}\;\left(\mathrm{SII}\right)={\left[\text{platelet}/\text{lymphocyte}\right]}^{*}\;\text{Neutrophil}$ ^34^
2)
$\text{Systemic Inflammation Response Index}\;\left(\text{SIRI}\right)={\left[\text{monocyte}/\text{lymphocyte}\right]}^{*}\;\text{Neutrophil}$ ^35^
3)
$\mathrm{Pan}\;\text{Immune Inflammation Value}\;\left(\mathrm{PIV}\right)={\text{Platelet}}^{*}\;{\left[\text{monocyte}/\text{lymphocyte}\right]}^{*}\;\text{Neutrophil}$ ^36^
4)
Neutrophil to Lymphocyte Ratio(NLR) ^10^
5)
Platelet to Lymphocyte Ratio(PLR) ^10^
6)
Monocyte to Lymphocyte Ratio(MLR) ^11^

The selected indices (SII, SIRI, PIV, NLR, MLR, PLR) are well-established markers of systemic inflammation and immune status. For instance, SII integrates platelet, neutrophil, and lymphocyte counts, reflecting the balance between inflammation and immune response. ^10, 11, 18^ These indices have been previously validated as prognostic or diagnostic markers in sepsis and neonatal inflammatory conditions. ^32, 34^ Therefore, they were chosen to comprehensively evaluate the inflammatory status in preterm neonates with sepsis.

IBM SPSS (Statistical Package for Social Sciences) Statistics for Windows Version 29.0. Armonk, NY:IBM Corp. used to analyse the data. Descriptive statistics were presented as standard deviations and means. An independent sample t-test was used to compare the scores of the case and control arms. Statistical significance was defined as a p-value of less than 0.05. To assess the predictive ability of the diagnostic markers, ROC (Receiver Operating Characteristic) analysis was performed. Additionally, the optimum cut-off value was determined using the Youden Index.

## Results

A total of 138 preterm neonates were included in this study, out of which, 50% of patients belonged to the sepsis group.

Table 1 shows the clinical characteristics of the neonates. Of the 69 infants in the control group, 49.3% were male. The babies were categorized as late preterm (52.2%), moderate preterm (18.8%), very preterm (27.5%), and extremely preterm (1.4%). The majority of babies were delivered via normal vaginal delivery (60.9%). There were 38 babies with low birth weight (LBW), 29 with very low birth weight (VLBW), one with extremely low birth weight (ELBW) baby and 1 baby of normal birth weight. 31.9 Of the babies, 31.9% were found to be small for gestational age (SGA) and 68.1% were found to be appropriate for gestational age (AGA).

**Table 1.** Maternal and neonatal characteristics of the study population. ^37^

Characteristic	Sepsis group (n = 69) n (%)	Control group (n = 69) n (%)
**Maternal age (years)**	26.46 ± 5.41	28.06 ± 4.86
**Sex of neonate**		
Male	35 (50.7)	34 (49.3)
Female	34 (49.3)	35 (50.7)
**Gestational age category**		
Late preterm (34–36 weeks)	23 (33.3)	36 (52.2)
Moderate preterm (32–33 weeks)	16 (23.2)	13 (18.8)
Very preterm (28–31 weeks)	29 (42.0)	19 (27.5)
Extremely preterm (<28 weeks)	1 (1.4)	1 (1.4)
**Mode of delivery**		
Vaginal delivery	39 (56.5)	42 (60.9)
Lower segment cesarean section (LSCS)	30 (43.5)	27 (39.1)
**Birth weight category**		
Low birth weight (LBW)	14 (20.3)	38 (55.1)
Very low birth weight (VLBW)	54 (78.3)	29 (42.0)
Extremely low birth weight (ELBW)	1 (1.4)	1 (1.4)
Normal birth weight (NBW)	0 (0.0)	1 (1.4)
**Growth status**		
Small for gestational age (SGA)	14 (20.3)	22 (31.9)
Appropriate for gestational age (AGA)	55 (79.7)	47 (68.1)

50.7 Of the patients in the sepsis group, 50.7% were male. Preterm sepsis was categorized as late preterm (33.3%), moderate preterm (23.3%), very preterm (42%), and extremely preterm (1.4%). None of the infants with sepsis had a normal birth weight. The majority of babies were delivered via normal vaginal delivery (56.5%). There were 14 LBW infants, 54 VLBW infants, and 1 ELBW infant. 20.3 Of the infants, 20.3% were small for gestational age (SGA) and 79.7% were appropriate for gestational age (AGA).

Table 2 shows laboratory parameters of the neonates. Compared to the control group, preterm babies of sepsis group had lower WBC, lower monocyte, lower neutrophil, lower platelet and lymphocyte counts. Values of platelet counts (P < 0.001), SII (P = 0.002), PIV (P < 0.001) and PLR (P < 0.001) were found to be statistically significant. Values of total count (P = 0.117), monocyte (P = 0.197), neutrophil (P = 0.694), lymphocyte (P = 0.436), SIRI (P = 0.944), NLR (P = 0.581) and MLR (P = 0.873) were not statistically significant.

**Table 2.** Laboratory parameters and inflammatory indices in preterm neonates with and without sepsis. ^37^

Parameter	Control (n = 69) Median (IQR)	Case (n = 69) Median (IQR)	P value
Total leukocyte count (cells/μL)	11,500 (8,650–16,300)	9,650 (6,610–16,380)	0.117
Monocytes (%)	8.0 (6.0–10.0)	6.0 (5.0–10.0)	0.197
Neutrophil (%)	59.0 (50.0–66.0)	56.0 (45.0–70.0)	0.694
Platelet count (cells/μl)	248,00 (202,000–299,000)	164,000 (70,000–218,000)	<0.001
Lymphocytes (%)	28.0 (21.0–36.0)	25.0 (17.0–36.0)	0.436
SII (Systemic Immune-Inflammatory Index)	496,909.9 (224,000.0–851,857.1)	232,400.0 (106,250.0–621,473.7)	0.002
SIRI (Systemic Inflammation Response Index)	16.0 (9.0–25.5)	14.9 (4.9–31.2)	0.944
PIV (Pan Immune Inflammation Value)	3,349,600.0 (1,503,673.5–6,404,363.6)	1,747,200.0 621,473.7–3,814,285.7)	<0.001
NLR (Neutrophil-to-Lymphocyte Ratio)	2.1 (1.5–3.2)	2.2 (1.3–3.8)	0.581
PLR (Platelet-to-Lymphocyte Ratio)	8,833.3 (4,939.4–12,866.7)	4,934.4 (2,588.2–9,803.3)	<0.001
MLR (Monocyte-to-Lymphocyte Ratio)	0.28 (0.17–0.40)	0.27 (0.14–0.48)	0.873

To evaluate the predictive power of SII, platelet count, PIV, and PLR for neonatal sepsis, the area under the curve (AUC) values were calculated from the receiver operating characteristic (ROC) curves. The cutoff points were determined using the Youden Index.

**Figure 2.**
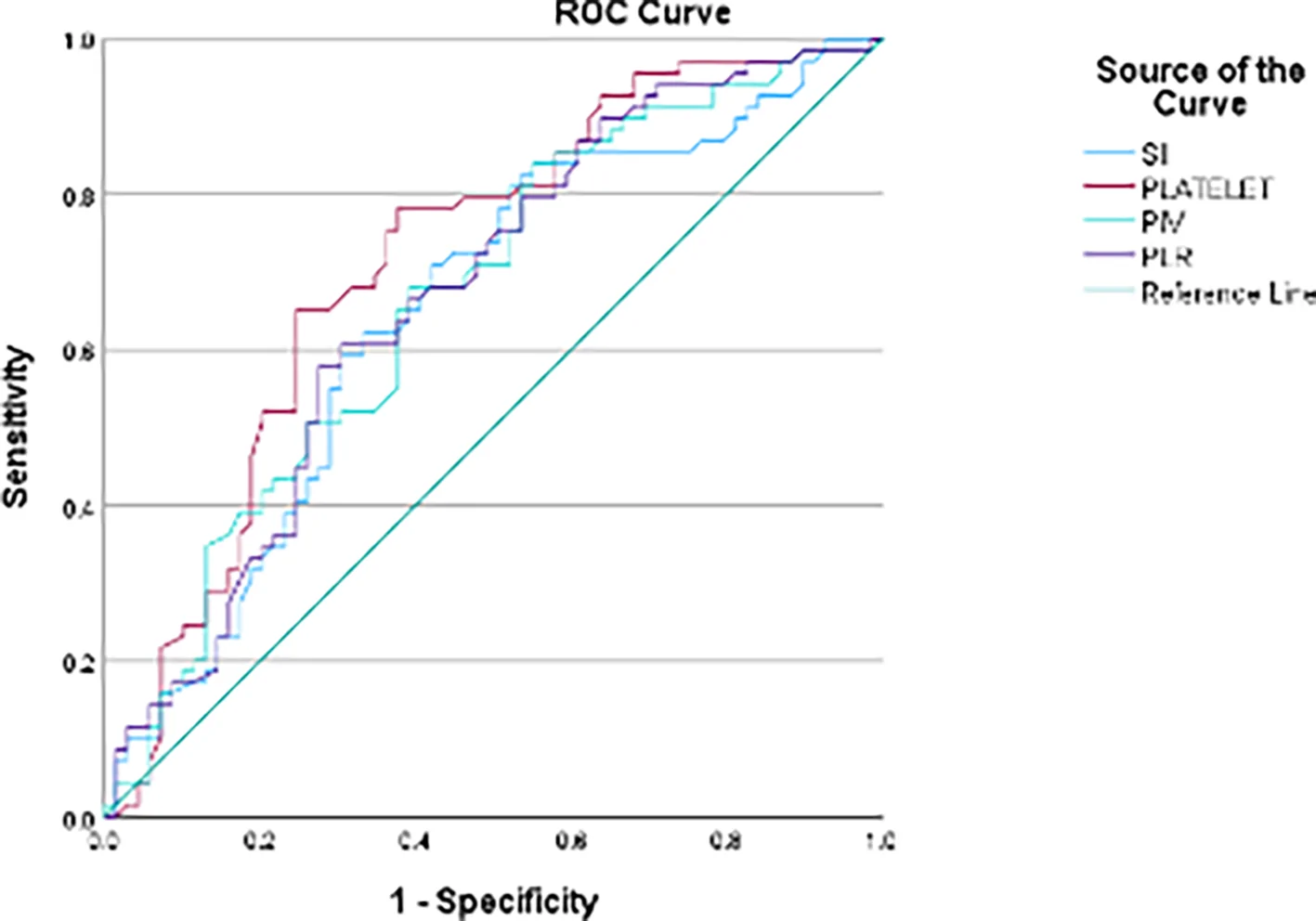
ROC curve analysis. ^37^

The ROC curves for the predictive ability of SII, platelet count, PIV, and PLR are shown in Figure 2. Tables 3 and 4 show the ROC analysis and optimal cutoff values. Of the four variables, platelet count had the highest AUC value (0.715), with an ideal cut-off concentrations of 219500 cells/cu mm and sensitivity and specificity of 75.4 and 65.2 respectively. The AUC value for PLR was 0.668, the sensitivity and specificity were 72.5 and 58%, respectively, and the ideal cut-off value was 7923.19. The AUC value and cut-off value for PIV were 0.665 and 2187333.33, respectively, with a sensitivity of 60.9 and specificity of 68.1. The AUC value was lowest for SII (0.65), with an ideal cut-off values of 37656.79, and sensitivity and specificity of 66.7 and 62.3%, respectively.

**Table 3.** ROC analysis to assess predictive ability of diagnostic markers to predict neonatal sepsis. ^37^

Parameter	AUC	p value	95% CI Lower Bound	95% CI Upper Bound
SII (Systemic Immune-Inflammatory Index)	0.65	0.002	0.558	0.743
Platelet count (cells/μL)	0.715	<0.001	0.628	0.802
PIV (Pan-Immune Inflammation Value)	0.665	0.001	0.574	0.755
PLR (Platelet-to-Lymphocyte Ratio)	0.668	0.001	0.577	0.758

**Table 4.** Optimal cutoff determined using ROC analysis. ^37^

Parameter	Cut-off	Sensitivity (%)	Specificity (%)
SII (Systemic Immune-Inflammatory Index)	37,656.79	66.7	62.3
Platelet count (cells/μL)	219,500	75.4	65.2
PIV (Pan-Immune Inflammation Value)	2,187,333.33	60.9	68.1
PLR (Platelet-to-Lymphocyte Ratio)	7,923.19	72.5	58.0

### Critical reflection

Of the 138 preterm babies included in the study, the majority were late preterm, 23 belonged to the sepsis group, and 36 belonged to the control group. Most of the babies in both the sepsis (56.5%) and control (60.9%) groups were delivered by normal vaginal delivery. The majority of babies in the sepsis group had very low birth weight (78.3%) and the majority in the control group had low birth weight (55.1%).

Platelet count, SII, PIV, and PLR were found to be significant predictors of neonatal sepsis. Platelet count had the highest predictive value, with an AUC value of 0.715 and optimal cut-off value of 219500 cells/cu mm. It had a sensitivity of 75.4 and specificity of 65.2.

## Discussion

Early detection of neonatal sepsis remains challenging due to nonspecific clinical signs and the time required for blood culture results. PLR, SII, and platelet count all showed differences between neonates with and without sepsis in our study, with platelet count exhibiting the strongest correlation. These findings are consistent with other research indicating that platelet-related indices may be useful in evaluating newborn sepsis. Day-one WBC and platelet counts could serve as early markers of sepsis, according to Xianghui Liang et al. and Minichil Worku et al. ^21, 23^ According to Isabelle M. C. Ree et al. and Vizcarra-Jimenez et al., ^24, 25^ thrombocytopenia has also been linked to higher mortality in neonatal sepsis, especially in gram-negative infections.

Previous research has also assessed inflammatory indices such SII, NLR, PLR, and SIRI. According to Zhu et al., newborn sepsis may be reflected by PLR, SII, and NLR, with SII exhibiting a comparatively larger predictive value. ^10^ Similarly SII, PLR, and NLR may also be useful in diagnosing sepsis-related outcomes, according to Mangalesh et al. and Islam et al. ^18, 20^ Higher SIRI and SII levels were linked to a higher risk of secondary infections in preterm newborns, according to Chen et al. ^17^ However, SIRI did not significantly differ in our study, suggesting that its effectiveness may differ depending on the population.

The potential application of SII in particular newborn diseases is supported by additional research. While Güngör et al and Runqiang Liang et al ^13, 14^ emphasized SII in urinary tract infections and serious bacterial infections respectively, Aydogan et al. reported that NLR and SII may be linked to outcomes in infants with congenital heart disease. ^12^ Preterm newborns with late-onset sepsis have increased SII levels, according to Vardar et al. ^28^ Furthermore, links between neonatal sepsis and hematologic abnormalities such as leukopenia, thrombocytopenia, anemia, and increased NLR were found by Mubaraki et al. and Li et al. ^29, 30^ These results imply that a variety of hematologic and inflammatory indices may be useful in predicting newborn sepsis, although the relative effectiveness of these indices may differ depending on the situation.

The absence of statistically significant difference in NLR unlike in most adult studies may reflect the unique hematological physiology of preterm neonates. Unlike adults, preterm infants have an immature neutrophil storage pool, resulting in attenuated neutrophilia. This blunted neutrophil response may reduce discriminatory capacity of NLR in this population. The AUC values reported in our study - ranging from 0.65 (SII) to 0.715(platelet count) represent moderate diagnostic performance. Hence these markers can have a role as supplementary indicators intended to complement early diagnosis, rather than used as standalone diagnostic tools.

It is important to note several limitations. The results of our study may not be generalizable to other groups because it was limited to a single tertiary care facility. The found associations may be influenced by variables like gestational age, birth weight, comorbidities, and laboratory techniques. Multivariate analysis and larger sample size will be required to establish independent predictive efficacy. To define uniform thresholds for clinical use and to further investigate the potential utility of these markers, larger multicenter trials are required.

Overall, our findings suggest that SII and PLR, as well as platelet count and thrombocyte-related indices, have substantial correlation with newborn sepsis. These findings support earlier research while emphasizing the need for careful interpretation and additional validation in larger groups.

### Limitations

This study was performed only among preterm infants in the NICU, and studies involving term infants admitted to the NICU may be required. Restricting cases and controls by culture results may have excluded subclinical or early infections, true infections and introduced misclassification. Studies involving culture negative sepsis will be required. Another major limitation of our study is that maternal factors such as chorioamnionitis, prolonged rupture of membranes, intrapartum antibiotic exposure, pre eclampsia and neonatal factors such as congenital anomalies, respiratory distress syndrome, postnatal corticosteroid or inotrope use, which may influence these indices were not controlled for. Multivariate analysis was not performed due to the limited sample size and absence of comprehensive covariate data at the time of analysis. Potential confounders such as gestational age and birth weight may have influenced the results. Further multicenter studies with larger patient populations and multivariate analysis will be required to determine the independent diagnostic utility of these markers.

## Conclusion

There was highest significant correlation between platelet count and positive cultures followed by PLR, PIV and SII, all with moderate statistical performance. Larger prospective trials should be conducted to further validate their potential clinical value. This will aid in early sepsis diagnosis and management and, in turn, reduce neonatal morbidity and mortality associated with sepsis.

## Ethical approval statement

On 17/10/24, ethical clearance was granted by The Institutional Ethics Committee at Kasturba Medical College in Mangalore (Protocol No: IECKMCMLR10/2024/606). The MS of the Government Lady Goschen Hospital has given us permission to conduct this study.

## Consent statement

As this was a laboratory record–based observational study utilizing routinely collected clinical and laboratory data without any additional sampling or intervention, individual informed consent was waived by the Institutional Ethics Committee of Kasturba Medical College, Mangalore.

## Data Availability

Figshare: Data – Excel Sheet (Neonatal sepsis research data Excel) https://doi.org/10.6084/m9.figshare.28395161.v1. ^37^ The project contains the following underlying data:
• Sepsis Data 2 Data are available under the terms of the Creative Commons Attribution 4.0 International license (CC-BY 4.0).
