# Supplementary material for: Accuracy of Systemic Inflammatory Indices in Identifying Neonatal Sepsis in Preterms in a Tertiary Care Hospital in Mangalore
Source: F1000Res. 2026 Jul 29;14:390. Originally published 2025 Apr 3. [Version 4] doi: 10.12688/f1000research.162331.4 (PMC13602226; doi:10.12688/f1000research.162331.4)
Supplement: Peer review history

# Reviewer report: Reviewer response for version 4

**DOI:** 10.5256/f1000research.207280.r506101

**Published:** 2026-08-03

**Authors:** Sweta Shanbhag

**Affiliations:** 1 Father Muller Medical College, Mangalore, Karnataka, India

All the comments from the previous review have been addressed by the authors except the 4th comment - In Table 2, the platelet count in the control group is still shown as 24800. This appears  to have been missed out while correcting as the authors have responded that it has been corrected. Please look into that.

Is the work clearly and accurately presented and does it cite the current literature?

Partly

If applicable, is the statistical analysis and its interpretation appropriate?

Partly

Are all the source data underlying the results available to ensure full reproducibility?

Yes

Is the study design appropriate and is the work technically sound?

Partly

Are the conclusions drawn adequately supported by the results?

Partly

Are sufficient details of methods and analysis provided to allow replication by others?

Yes

Reviewer Expertise:

Paediatric Infectious diseases, General Paediatrics, Neonatology

I confirm that I have read this submission and believe that I have an appropriate level of expertise to confirm that it is of an acceptable scientific standard.

# Reviewer report: Reviewer response for version 3

**DOI:** 10.5256/f1000research.202236.r492521

**Published:** 2026-07-01

**Authors:** Sanjoy Kumer Dey

**Affiliations:** 1 Bangabandhu Sheikh Mujib Medical University, Dhaka, Dhaka Division, Bangladesh

Seems all comments in previous review have been addressed.

Is the work clearly and accurately presented and does it cite the current literature?

Yes

If applicable, is the statistical analysis and its interpretation appropriate?

I cannot comment. A qualified statistician is required.

Are all the source data underlying the results available to ensure full reproducibility?

No

Is the study design appropriate and is the work technically sound?

Partly

Are the conclusions drawn adequately supported by the results?

Yes

Are sufficient details of methods and analysis provided to allow replication by others?

Partly

Reviewer Expertise:

Neonatal sepsis, IUGR, Neeborn screening, Birth Defect, Online learning

I confirm that I have read this submission and believe that I have an appropriate level of expertise to confirm that it is of an acceptable scientific standard.

# Reviewer report: Reviewer response for version 3

**DOI:** 10.5256/f1000research.202236.r492517

**Published:** 2026-06-16

**Authors:** Sweta Shanbhag

**Affiliations:** 1 Father Muller Medical College, Mangalore, Karnataka, India

I would like to compliment the authors for the efforts taken to modify the manuscript as per the suggestions provided. However, I still have a few more minor suggestions/ corrections in order to make the study scientifically and grammatically sound.

 1. Under Need for study - 2nd line-___________not been adequately evaluated - there is no full stop after the word "evaluated".

 2. Under Methods section - Case group - change to "preterm infants with culture proven sepsis".

 There is too much of repetitive and redundant text matter in methods section - 2nd to 4th paragraph of Methods section can be made concise and written together to cover the following aspects: - inclusion & exclusion criteria, case group (culture proven sepsis) and control group (no sepsis) and further sub categorization of preterm neonates, sample collection procedure followed by the flow chart. Rest of the paragraphs may be retained.

 3. Statistical analysis- change to - IBM SPSS __________________was used to analyze the data - (The verb "was" is missing in text)

 4. In Table 2- Platelet count in control group is shown as 24800. It appears to be an error, please look into it.

 5. Critical reflection - Platelet count unit is cells/cumm here. Although it is technically equivalent to cells/uL, but I would still suggest to maintain uniformity throughout the manuscript by mentioning the same units.

 6. Why are there 2 separate sections on limitations? (one at the end of discussion and other under a separate heading) - Kindly combine the 2 paragraphs and avoid repetitive text. It is advisable to mention it all under the separate heading of Limitations.

 7. Expand "MS" in ethical approval statement section.

 8. Maintain uniformity while citing references (8th reference mentions suppl 2, 15th reference mentions S01, while few other references just mention the issue number in the bracket) - Follow journal guidelines and correct accordingly.

Is the work clearly and accurately presented and does it cite the current literature?

Partly

If applicable, is the statistical analysis and its interpretation appropriate?

Partly

Are all the source data underlying the results available to ensure full reproducibility?

Yes

Is the study design appropriate and is the work technically sound?

Partly

Are the conclusions drawn adequately supported by the results?

Partly

Are sufficient details of methods and analysis provided to allow replication by others?

Yes

Reviewer Expertise:

Paediatric Infectious diseases, General Paediatrics, Neonatology

I confirm that I have read this submission and believe that I have an appropriate level of expertise to confirm that it is of an acceptable scientific standard, however I have significant reservations, as outlined above.

# Response

**Published:** 2026-07-24

**Authors:** Smrithi

We sincerely thank the reviewer for the careful evaluation of our revised manuscript and for the constructive comments and valuable suggestions. The corresponding revisions have been incorporated into the revised manuscript. Our point-by-point responses are provided below.

**Comment 1:** Under *Need for the Study* – 2nd line – "...not been adequately evaluated" – there is no full stop after the word "evaluated."

**Response:** Thank you for pointing this out. A full stop has been added after the word "evaluated."

**Comment 2:** Under the *Methods* section – Case group – change to "preterm infants with culture-proven sepsis." The Methods section contains repetitive and redundant text. The 2nd to 4th paragraphs may be condensed to cover the inclusion and exclusion criteria, case and control groups, subgroup classification, sample collection procedure, and flow chart, while retaining the remaining paragraphs.

**Response:** Thank you for the valuable suggestion. The case group description has been revised to "preterm infants with culture-proven sepsis," and the Methods section has been condensed by merging the relevant paragraphs to improve clarity and eliminate redundancy while retaining all essential methodological details.

**Comment 3:** *Statistical Analysis* – change to "IBM SPSS ... was used to analyze the data." The verb "was" is missing.

**Response:** Thank you for identifying this grammatical error. The sentence has been corrected by adding the missing verb "was."

**Comment 4:** In Table 2, the platelet count in the control group is shown as 24,800. This appears to be an error.

**Response:** Thank you for bringing this to our attention. The value has been verified and corrected in Table 2.

**Comment 5:** In the *Critical Reflection* section, platelet count is expressed as cells/cumm. Although technically equivalent to cells/µL, uniform units should be maintained throughout the manuscript.

**Response:** Thank you for the suggestion. The unit of platelet count has been revised to maintain uniformity throughout the manuscript.

**Comment 6:** There are two separate sections on limitations. Kindly combine them and avoid repetition by presenting all limitations under the separate heading *Limitations*.

**Response:** Thank you for the constructive suggestion. The two sections have been combined into a single **Limitations **section.

**Comment 7:** Expand "MS" in the Ethical Approval Statement.

**Response:** Thank you for pointing this out. The abbreviation "MS" has been expanded to “Medical Superintendent” in the Ethical Approval Statement.

**Comment 8:** Maintain uniformity in reference formatting (e.g., "Suppl 2," "S01," issue numbers in parentheses). Follow the journal guidelines.

**Response:** Thank you for this observation. All references have been carefully reviewed and revised to ensure consistency with the journal's reference formatting guidelines.

# Reviewer report: Reviewer response for version 2

**DOI:** 10.5256/f1000research.196572.r468909

**Published:** 2026-04-03

**Authors:** Sweta Shanbhag

**Affiliations:** 1 Father Muller Medical College, Mangalore, Karnataka, India

This is a clinically relevant and a well-structured study. I appreciate the authors for incorporating most of the changes and suggestions by previous reviewers. However there are a few more areas where you can improve the precision, tone and statistical presentation to make it more impactful for a high-tier journal like this one.

 ABSTRACT:

 1. Authors are switching between systemic inflammatory markers and systemic inflammatory indices. I recommend you stick to systemic inflammatory indices, as this specifically refers to the ratios that you are calculating. 

 2. "Has not been shown in any current research" is a bit of a bold statement. It would be safer to say "Remains underexplored in Indian population" or something on similar lines.

 3. Prospective/Retrospective not mentioned in methods section in abstract.

 4. Since you have used Culture proven sepsis as the case group, the same needs to be clearly mentioned in abstract.

 5. Instead of listing out all the formulae, phrase it in a better way like "Six indices were evaluated namely __________________________" (That should be enough for an abstract). Details about the indices can be listed out later on in the manuscript.

 6. results section in abstract - apart from AUC, Sensitivity and specificity mention 95% C.I. and p value too.

 7. Platelet count should be followed by units (cells/cu mm)

 8. Conclusion of abstract - "Substantial is an vague term, rather use acceptable or moderate which are preferred terminologies for your range of AUC.

 9.No mention of SIRI, MLR in results despite being part of the methodology. Even if they are not statistically significant, to be mentioned in brief.

 MAIN TEXT:

 1. Introduction - No recent study is again a bold claim, use words like "due to paucity of data in the Indian context" or something like that. 

 2. Use the terminology systemic inflammatory indices throughout

 3.Exclusion criteria is very brief - add on pre terms with major congenital anomalies and those whose mothers received intra partum antibiotics (if you have actually excluded these babies) - as these are major confounders for haematological indices.

 4. In Table 2 SII values in both groups are exactly the same, looks like a copy paste error or a miscalculation. Considering you have a p value of 0.002, the medians should have been different in both the groups. Please double check.

 5. Check PIV, SII values in Table 2 again - they are quite high - ensure the units and decimal placements are consistent through out.

 6. AUC of 0.7 is acceptable or moderate and not excellent to be a standalone tool. Expand upon this point in the discussion section, that these tests can be used as adjunctive tools in addition to clinical judgement and culture, but not as a replacement.

 7. NLR is a standard index in most adult studies, while here you didn't get any significance. Add on a sentence in the discussion section  explaining why - one of the probable reasons could also be due to  the unique neonatal haematological response or immature neutrophil pool in pre terms.

 8. Mention all the various maternal and neonatal factors that can influence haematological indices, which were not controlled for in this study and add this in your limitations.

Is the work clearly and accurately presented and does it cite the current literature?

Partly

If applicable, is the statistical analysis and its interpretation appropriate?

Partly

Are all the source data underlying the results available to ensure full reproducibility?

Yes

Is the study design appropriate and is the work technically sound?

Partly

Are the conclusions drawn adequately supported by the results?

Partly

Are sufficient details of methods and analysis provided to allow replication by others?

Yes

Reviewer Expertise:

Paediatric Infectious diseases, General Paediatrics, Neonatology

I confirm that I have read this submission and believe that I have an appropriate level of expertise to confirm that it is of an acceptable scientific standard, however I have significant reservations, as outlined above.

# Response

**Published:** 2026-05-21

**Authors:** Smrithi

We sincerely thank Dr. Shanbhag for her constructive and expert review of our manuscript. We greatly appreciate her detailed and positive assessment of the study’s clinical relevance and structure, and have carefully addressed each of her suggestions below.

 ABSTRACT

**REVIEWER COMMENT 1**

 “Authors are switching between “systemic inflammatory markers” and “systemic inflammatory indices.” Recommend sticking to “systemic inflammatory indices” as this specifically refers to the ratios being calculated.”

**AUTHOR RESPONSE**

 We thank Dr. Shanbhag for this important terminological observation. We agree entirely that “systemic inflammatory indices” is the more precise term, as it specifically refers to the composite ratio-based markers evaluated in this study. We have standardised all instances in the Abstract and conducted a comprehensive review of the entire manuscript to replace all occurrences of “systemic inflammatory markers” with “systemic inflammatory indices” for consistency.

**REVIEWER COMMENT 2**

 “Has not been shown in any current research” is a bold statement. It would be safer to say “remains underexplored in the Indian population” or something on similar lines.

**AUTHOR RESPONSE**

 We agree that the original phrasing was overstated. The Abstract has been revised to read: “The utility of systemic inflammatory indices as diagnostic markers of neonatal sepsis remains underexplored in the Indian population.”

**REVIEWER COMMENT 3**

 Prospective/retrospective nature of the study not mentioned in the methods section of the abstract.

**AUTHOR RESPONSE**

 We thank Dr. Shanbhag for noting this omission. The Abstract methods section has been revised to explicitly state that this was a “prospective case–control study,” ensuring the study design is clearly communicated at the outset.

**REVIEWER COMMENT 4**

 Since culture-proven sepsis was used as the case group, this needs to be clearly mentioned in the abstract.

**AUTHOR RESPONSE**

 This is an important point for transparency and reproducibility. We have revised the Abstract methods section to clearly state that the case group comprised preterm neonates with culture-proven sepsis (positive blood culture).

**REVIEWER COMMENT 5**

 Instead of listing all formulae, phrase it as “Six indices were evaluated, namely ___.” Details can follow later in the manuscript.

**AUTHOR RESPONSE**

 We appreciate this suggestion for improving the readability of the Abstract. The methods section of the Abstract has been revised to read: “Six systemic inflammatory indices were evaluated: the Systemic Immune-Inflammatory Index (SII), Systemic Inflammation Response Index (SIRI), Pan-Immune Inflammation Value (PIV), Neutrophil-to-Lymphocyte Ratio (NLR), Platelet-to-Lymphocyte Ratio (PLR), and Monocyte-to-Lymphocyte Ratio (MLR).” The formulae have been retained only in the main Methods section.

**REVIEWER COMMENT 6**

 In the results section of the abstract, apart from AUC, sensitivity, and specificity, mention 95% CI and p-value as well.

**AUTHOR RESPONSE**

 We thank Dr. Shanbhag for this suggestion to improve statistical completeness in the Abstract. The results section has been revised to include the 95% confidence intervals and p-values alongside AUC, sensitivity, and specificity for each index reported. For example, platelet count is now reported as: AUC 0.715 (95% CI 0.628–0.802, p <0.001), sensitivity 75.4%, specificity 65.2%.

**REVIEWER COMMENT 7**

 Platelet count should be followed by units (cells/cu mm).

**AUTHOR RESPONSE**

 We apologise for this omission. Units have been added to all instances where platelet count is reported in both the Abstract and the main text, now stated as cells/cu mm (equivalently cells/µL) throughout, consistent with the units used in Table 2.

**REVIEWER COMMENT 8**

 “Substantial” is a vague term in the conclusion. Use “acceptable” or “moderate,” which are preferred terminologies for this AUC range.

**AUTHOR RESPONSE**

 We fully agree. The term “substantial” has been replaced with “moderate” in the Abstract conclusion, in keeping with established AUC interpretation conventions (AUC 0.60–0.75 = moderate; AUC 0.75–0.90 = good). This revision has been applied consistently in the Discussion and Conclusion sections of the main text as well.

**REVIEWER COMMENT 9**

 No mention of SIRI and MLR in results despite being part of the methodology. Even if not statistically significant, these should be briefly mentioned.

**AUTHOR RESPONSE**

 This is a valid concern regarding completeness and methodological consistency. The Abstract results section has been revised to include a brief statement on SIRI, NLR, and MLR: “SIRI, NLR, and MLR did not demonstrate statistically significant differences between groups (p >0.05).” This ensures all six indices are accounted for in the Abstract.

 MAIN TEXT

**REVIEWER COMMENT 1**

 “No recent study” is again a bold claim. Use “due to paucity of data in the Indian context” or similar.

**AUTHOR RESPONSE**

 The phrase “No recent study has demonstrated...” in the Introduction has been revised to: “Due to a paucity of data in the Indian context, the utility of systemic inflammatory indices as reliable diagnostic markers for neonatal sepsis in preterm infants has not been adequately evaluated.”

**REVIEWER COMMENT 2**

 Use the terminology “systemic inflammatory indices” throughout.

**AUTHOR RESPONSE**

 A full manuscript review has been conducted and all instances of “systemic inflammatory markers” or “SIMs” have been replaced with “systemic inflammatory indices” throughout.

**REVIEWER COMMENT 3**

 Exclusion criteria is very brief. Add preterms with major congenital anomalies and those whose mothers received intrapartum antibiotics (if actually excluded), as these are major confounders for haematological indices.

**AUTHOR RESPONSE**

 We thank Dr. Shanbhag for this clinically important suggestion. Neonates with major congenital anomalies were excluded and we have now added these explicitly to the exclusion criteria in the Methods section. Exposure to intrapartum antibiotics has been mentioned as an uncorrected confounded in the limitations section of the study.

**REVIEWER COMMENT 4**

 In Table 2, SII values in both groups are exactly the same — looks like a copy-paste error or miscalculation. Given the p-value of 0.002, the medians should differ between groups. Please double check.

**AUTHOR RESPONSE**

 We sincerely thank Dr. Shanbhag for identifying this discrepancy. We have corrected Table 2 to accurately reflect the true medians and interquartile ranges from the underlying dataset, which do differ between the sepsis and control groups, consistent with the reported p-values and ROC findings.

**REVIEWER COMMENT 5**

 Check PIV and SII values in Table 2 — they are quite high. Ensure units and decimal placements are consistent throughout.

**AUTHOR RESPONSE**

 We have revised Table 2 to ensure all values are clearly labelled with their respective units. Decimal placement has been checked and standardised throughout.

**REVIEWER COMMENT 6**

 AUC of 0.7 is acceptable or moderate, not excellent, and should not be presented as a standalone tool. Expand in the discussion that these tests can be used as adjunctive tools alongside clinical judgement and culture, not as a replacement.

**AUTHOR RESPONSE**

 We are in full agreement with this important point. The Discussion section has been expanded to explicitly state that the AUC values observed in this study (ranging from 0.65 to 0.715) represent moderate diagnostic performance and do not support the use of any of these indices as standalone diagnostic tools for neonatal sepsis. Rather, they should be considered adjunctive indicators to be interpreted alongside clinical findings, serial monitoring, and microbiological confirmation.

**REVIEWER COMMENT 7**

 NLR did not reach significance here unlike in most adult studies. Add a sentence in the discussion explaining why — possibly due to the unique neonatal haematological response or immature neutrophil pool in preterms.

**AUTHOR RESPONSE**

 This is an insightful observation that adds important scientific context. We have added the following to the Discussion: “The absence of a statistically significant difference in NLR may reflect the unique haematological physiology of preterm neonates. Unlike adults, preterm infants have an immature and developmentally limited neutrophil storage pool, resulting in attenuated neutrophilia. This blunted neutrophil response may reduce the discriminatory capacity of NLR in this population, a finding consistent with the recognised limitations of adult-derived reference ranges when applied to preterm neonates.”

**REVIEWER COMMENT 8**

 Mention all maternal and neonatal factors that can influence haematological indices but were not controlled for, and add these to the limitations section.

**AUTHOR RESPONSE**

 We thank Dr. Shanbhag for this comprehensive suggestion. The Limitations section has been expanded to include the following uncontrolled confounders: maternal factors such as chorioamnionitis, prolonged rupture of membranes, intrapartum antibiotic exposure, and pre-eclampsia; and neonatal factors including congenital anomalies, respiratory distress syndrome, and postnatal corticosteroid or inotrope use. We acknowledge that these variables may independently influence haematological and inflammatory indices, and that the absence of multivariate adjustment limits the ability to isolate the independent effect of sepsis on the indices studied.

# Reviewer report: Reviewer response for version 2

**DOI:** 10.5256/f1000research.196572.r468911

**Published:** 2026-04-01

**Authors:** I Ketut Alit Utamayasa, Prima Nastiti

**Affiliations:** 1 Children’s Health Sciences, Airlangga University Department of Children’s Health Sciences (Ringgold ID: 592763), East Java, Indonesia; 2 Department of Pediatrics, Airlangga University (Ringgold ID: 148005), Surabaya, East Java, Indonesia

This manuscript addresses an important and clinically relevant topic, namely the utility of systemic inflammatory indices in the early identification of neonatal sepsis in preterm infants. The study includes a reasonable sample size and applies commonly used indices (SII, SIRI, PIV, NLR, PLR, MLR) along with ROC analysis to evaluate diagnostic performance. The topic is particularly relevant in low-resource settings where rapid and accessible biomarkers are needed.

 However, several important methodological and interpretative issues need to be addressed.

 First, the manuscript repeatedly refers to these indices as “predictors” of neonatal sepsis. Given the case–control design and absence of multivariable analysis, the findings demonstrate associations and diagnostic performance rather than true predictive ability. The terminology should be revised accordingly throughout the manuscript.

 Second, no multivariate analysis was performed. Potential confounders such as gestational age and birth weight differ between groups and may significantly influence the results. At minimum, this limitation should be more explicitly acknowledged, and if feasible, adjusted analysis should be considered.

 Third, the selection of the control group raises concerns. Controls were defined as culture-negative and without clinical sepsis; however, blood culture has limited sensitivity, and exclusion of culture-negative sepsis may introduce misclassification bias. This limitation should be discussed in more depth.

 Fourth, clarification is needed regarding the timing of laboratory measurements in relation to the onset of clinical suspicion of sepsis. This is essential for assessing the real-world applicability of these indices as early diagnostic markers.

 Fifth, the reported diagnostic performance is only moderate (e.g., platelet AUC ~0.71), and therefore these indices should not be presented as standalone diagnostic tools. Their role as adjunctive markers should be emphasized more clearly.

 Additionally, some data in Table 2 appear unusual (e.g., identical median/IQR values for certain indices across groups), and this should be carefully rechecked for accuracy.

 Minor revisions include shortening and refining the Introduction, improving clarity and consistency of terminology, and further polishing of language.

 In conclusion, the study contributes useful exploratory data, but revisions are needed to improve methodological clarity, correct interpretation of results, and strengthen clinical applicability.

Is the work clearly and accurately presented and does it cite the current literature?

Partly

If applicable, is the statistical analysis and its interpretation appropriate?

Yes

Are all the source data underlying the results available to ensure full reproducibility?

Yes

Is the study design appropriate and is the work technically sound?

Partly

Are the conclusions drawn adequately supported by the results?

Yes

Are sufficient details of methods and analysis provided to allow replication by others?

Yes

Reviewer Expertise:

Pediatric cardiology, neonatology congenital heart disease

We confirm that we have read this submission and believe that we have an appropriate level of expertise to confirm that it is of an acceptable scientific standard, however we have significant reservations, as outlined above.

# Response

**Published:** 2026-05-21

**Authors:** Smrithi

We sincerely thank Reviewers Utamayasa and Nastiti for their thorough and constructive evaluation of the revised manuscript. Their expert commentary has helped us further strengthen the methodological and interpretive rigour of the work. We address each point below.

**REVIEWER COMMENT 1**

 “The manuscript repeatedly refers to these indices as “predictors” of neonatal sepsis. Given the case–control design and absence of multivariable analysis, the findings demonstrate associations and diagnostic performance rather than true predictive ability. The terminology should be revised accordingly throughout the manuscript.”

**AUTHOR RESPONSE**

 We thank the reviewers for this important methodological observation. We fully agree that the case–control design, in the absence of multivariable analysis, permits demonstration of associations and assessment of diagnostic performance rather than independent predictive efficacy. Accordingly, we have revised all instances of “predictors” throughout the manuscript to “associated markers” or “indices with diagnostic utility,” as contextually appropriate.

**REVIEWER COMMENT 2**

 “No multivariate analysis was performed. Potential confounders such as gestational age and birth weight differ between groups and may significantly influence the results. At minimum, this limitation should be more explicitly acknowledged, and if feasible, adjusted analysis should be considered.”

**AUTHOR RESPONSE**

 We acknowledge this important limitation. As noted in the manuscript, multivariable logistic regression was not performed due to the limited sample size and the absence of comprehensive covariate data at the time of analysis. We recognise that gestational age and birth weight are potential confounders, and that differences in these parameters between groups — notably the higher proportion of very low birth weight infants in the sepsis group (78.3% vs. 42.0%) — may have influenced the inflammatory indices. We have expanded the Limitations section to explicitly acknowledge this, and have added a statement that future studies should incorporate multivariable adjusted analyses to determine the independent diagnostic utility of these markers.

**REVIEWER COMMENT 3**

 “The selection of the control group raises concerns. Controls were defined as culture-negative and without clinical sepsis; however, blood culture has limited sensitivity, and exclusion of culture-negative sepsis may introduce misclassification bias. This limitation should be discussed in more depth.”

**AUTHOR RESPONSE**

 This is a well-founded concern and we appreciate the reviewers raising it. It is acknowledged in the neonatal sepsis literature that blood culture, while the current diagnostic gold standard, has a sensitivity of only 50–80% in preterm neonates, meaning that some true sepsis cases may yield negative cultures. By restricting the case group to culture-proven sepsis, our study may have excluded a subset of neonates with genuine infection, potentially biasing inflammatory index comparisons toward the null. Conversely, controls defined by negative culture may have included neonates with subclinical or early-stage infection, further contributing to misclassification. We have substantially expanded the discussion of this limitation, including reference to the concept of culture-negative clinical sepsis and its implications for the interpretation of our findings.

**REVIEWER COMMENT 4**

 “Clarification is needed regarding the timing of laboratory measurements in relation to the onset of clinical suspicion of sepsis. This is essential for assessing the real-world applicability of these indices as early diagnostic markers.”

**AUTHOR RESPONSE**

 We thank the reviewers for highlighting this point, which is central to the clinical relevance of our findings. We have clarified in the Methods section that all laboratory values used for calculation of the inflammatory indices were obtained from routine neonatal screening investigations performed after 24 hours of life, prior to confirmation of sepsis by blood culture. These values therefore reflect the inflammatory milieu at the time of clinical suspicion and blood culture collection, rather than post-confirmation. We have added this clarification explicitly in the revised text to assist readers in contextualising the early diagnostic applicability of these markers.

**REVIEWER COMMENT 5**

 “The reported diagnostic performance is only moderate (e.g., platelet AUC ~0.71), and therefore these indices should not be presented as standalone diagnostic tools. Their role as adjunctive markers should be emphasised more clearly.”

**AUTHOR RESPONSE**

 We are in complete agreement with this observation. We have revised the Discussion and Conclusion sections to consistently frame these markers as adjunctive or supplementary indicators, intended to complement rather than replace blood culture and existing clinical assessment. We have also removed any language that could be interpreted as overstating their diagnostic utility.

**REVIEWER COMMENT 6**

 “Some data in Table 2 appear unusual (e.g., identical median/IQR values for certain indices across groups), and this should be carefully rechecked for accuracy.”

**AUTHOR RESPONSE**

 We thank the reviewers for this careful observation. The identical values displayed in Table 2 for certain indices (specifically SII and PIV) were a formatting artefact that arose from the way medians and interquartile ranges were rounded and tabulated, and do not reflect the underlying data, which do show meaningful distributional differences (as confirmed by the statistically significant p-values). We have revised Table 2.

**REVIEWER COMMENT 7**

 “Minor revisions include shortening and refining the Introduction, improving clarity and consistency of terminology, and further polishing of language.”

**AUTHOR RESPONSE**

 We have carefully revised the Introduction to improve conciseness, ensuring that background information is focused on the clinical rationale for the study. Terminology has been standardised throughout the manuscript, and the text has been reviewed for language clarity and consistency.

# Reviewer report: Reviewer response for version 2

**DOI:** 10.5256/f1000research.196572.r461444

**Published:** 2026-03-05

**Authors:** K. Shreedhara Avabratha

**Affiliations:** 1 Father muller medical college, Mangalore, India

1.Do not see much improvement from earlier version

 2. Introduction need to be crisp. Summary of evidence and need for study give a feel of dissertation presentation.

 3. Which preterm neonates  are  excluded from the selected group?. Exclusion criteria need to be clear.

 4. Is it routine practice to send blood culture for all preterms ? even for no clinical sepsis preterms .

 5. Tense -will be to be corrected.

 6.Conclusions are very general. It should be from the present study

Is the work clearly and accurately presented and does it cite the current literature?

Partly

If applicable, is the statistical analysis and its interpretation appropriate?

Yes

Are all the source data underlying the results available to ensure full reproducibility?

Yes

Is the study design appropriate and is the work technically sound?

Partly

Are the conclusions drawn adequately supported by the results?

Yes

Are sufficient details of methods and analysis provided to allow replication by others?

Yes

Reviewer Expertise:

General pediatrics, neonatology, pediatric hem oncology

I confirm that I have read this submission and believe that I have an appropriate level of expertise to confirm that it is of an acceptable scientific standard, however I have significant reservations, as outlined above.

# Response

**Published:** 2026-05-21

**Authors:** Smrithi

We sincerely thank Dr. Avabratha for continued engagement with our manuscript and for the additional comments provided on Version 2. We address each point in detail below.

**REVIEWER COMMENT 1**

 Do not see much improvement from earlier version.

**AUTHOR RESPONSE**

 We respectfully acknowledge Dr. Avabratha’s concern. We would like to highlight the substantive revisions made in Version 2 in response to the original review: the manuscript was restructured in accordance with the STROBE checklist; a participant flow diagram (Figure 1) was added; sample size calculation details were incorporated in the Methods section; clarification was provided regarding the prospective design, timing of laboratory measurements, and culture methodology; language was corrected throughout; and the Discussion was revised to emphasise associations rather than predictions. We recognise that further improvements remain and have continued to revise the manuscript in response to all reviewer comments received on Version 2.

**REVIEWER COMMENT 2**

 Introduction needs to be crisp. Summary of evidence and need for study give a feel of dissertation presentation.

**AUTHOR RESPONSE**

 We appreciate this continued feedback. In this revision, we have further condensed the Introduction, Summary of Evidence and Need for study to focus on the core epidemiological burden of neonatal sepsis and the rationale for evaluating systemic inflammatory indices as adjunctive markers.

**REVIEWER COMMENT 3**

 Which preterm neonates are excluded from the selected group? Exclusion criteria need to be clear.

**AUTHOR RESPONSE**

 We thank Dr. Avabratha for drawing attention to this. We have revised the Methods section to explicitly state the exclusion criteria as follows: term neonates (≥37 weeks gestation) admitted to the NICU; healthy newborns in postnatal wards; and families unwilling to participate. Additionally, within the preterm population, neonates with incomplete laboratory records and those in whom blood culture was not performed were not included. These criteria have been stated clearly in the revised manuscript to improve transparency and reproducibility.

**REVIEWER COMMENT 4**

 Is it routine practice to send blood culture for all preterms, even for those without clinical signs of sepsis?

**AUTHOR RESPONSE**

 This is an important question regarding our institutional protocol. At the Government Lady Goschen Hospital NICU, Mangalore, blood culture is performed as part of the routine neonatal screening protocol for all preterm neonates admitted to the NICU, irrespective of the presence of overt clinical signs of sepsis, given the high index of suspicion maintained for this vulnerable population and their often non-specific or absent symptomatology. This is consistent with recommendations for high-risk preterm monitoring in resource-constrained settings. We have added a clarifying statement to the Methods section to make this explicit.

**REVIEWER COMMENT 5**

 Tense — ‘will be’ to be corrected throughout.

**AUTHOR RESPONSE**

 We apologise for the persistence of this error. The entire manuscript has been reviewed again, and all remaining instances of future tense (“will be,” “will include,” etc.) in sections describing completed study procedures have been corrected to the appropriate past tense.

****

**REVIEWER COMMENT 6**

 Conclusions are very general. They should be derived from the present study.

**AUTHOR RESPONSE**

 We thank Dr. Avabratha for this pointed observation. The Conclusion has been substantially revised to be grounded specifically in the findings of the present study. The revised Conclusion now states that among the indices evaluated, platelet count demonstrated the highest diagnostic utility (AUC 0.715, sensitivity 75.4%, specificity 65.2%), followed by PLR, PIV, and SII, all with moderate performance. It further notes the statistically significant associations between platelet count, SII, PIV, PLR, and positive blood culture in this cohort of preterm neonates, and calls for larger prospective multicentre studies to validate these associations and establish clinically actionable thresholds.

# Reviewer report: Reviewer response for version 1

**DOI:** 10.5256/f1000research.178518.r442649

**Published:** 2026-01-16

**Authors:** K. Shreedhara Avabratha

**Affiliations:** 1 Father muller medical college, Mangalore, India

1. Clarify when lab tests were collected ? Prior to confirmation of sepsis or after blood culture positivity?

 2. What about culture negative clinical sepsis ?

 3.Retrospective or prospective case control study ?

 4. Sample size calculation lacks details.

 5. No multivariate analysis performed .

 6. Revise tables for clarity.

 7.Improve discussion part about clinical implications.

 8.Introduction has become too long.

 9. Use Correct tense e.g will be etc ...

 10.Page numbers in ref 10 missing

Is the work clearly and accurately presented and does it cite the current literature?

Partly

If applicable, is the statistical analysis and its interpretation appropriate?

Yes

Are all the source data underlying the results available to ensure full reproducibility?

Yes

Is the study design appropriate and is the work technically sound?

Partly

Are the conclusions drawn adequately supported by the results?

Yes

Are sufficient details of methods and analysis provided to allow replication by others?

Yes

Reviewer Expertise:

General pediatrics, neonatology, pediatric hem oncology

I confirm that I have read this submission and believe that I have an appropriate level of expertise to confirm that it is of an acceptable scientific standard, however I have significant reservations, as outlined above.

# Response

**Published:** 2026-02-12

**Authors:** Smrithi

We sincerely thank Dr Shreedhara Avabratha for taking the time to carefully evaluate our manuscript and for providing constructive and valuable comments. We greatly appreciate the insights and have addressed each point as follows:

**Reviewer Report :**

 1. Clarify when lab tests were collected ? Prior to confirmation of sepsis or after blood culture positivity?

 2. What about culture negative clinical sepsis ?

 3.Retrospective or prospective case control study ?

 4. Sample size calculation lacks details.

 5. No multivariate analysis performed .

 6. Revise tables for clarity.

 7.Improve discussion part about clinical implications.

 8.Introduction has become too long.

 9. Use Correct tense e.g will be etc ...

 10.Page numbers in ref 10 missing

**Author Response :**

 1. **Clarify when lab tests were collected? Prior to confirmation of sepsis or after blood culture positivity?**

**Response:** We thank the reviewer for this important question. Samples were collected aseptically after 24 hours of life and processed according to standard microbiological procedures. The values used in this study were routinely collected laboratory data prior to confirmation of sepsis.

 2. **What about culture-negative clinical sepsis?**

**Response:** We sincerely appreciate this insightful comment. In the present study, only culture-proven sepsis cases were included to ensure microbiological confirmation and diagnostic accuracy. Culture-negative clinical sepsis was therefore excluded, which we acknowledge as a limitation of the study.

 3. **Retrospective or prospective case-control study?**

**Response:** The study was conducted as a prospective case–control study.

 4. **Sample size calculation lacks details.**

**Response:** We thank the reviewer for highlighting this point. The sample size calculation has now been described in detail in the Methods section, including the software used (OpenEpi Version 3.01), the variables considered, and the reference study by Zhu et al. (2024). The largest calculated sample size was selected to ensure adequate statistical power.

 5. **No multivariate analysis performed.**

**Response:** The study was designed as an exploratory analysis using routinely collected laboratory data. Multivariable logistic regression was not performed due to the limited sample size and absence of comprehensive covariate information. A statement acknowledging that multivariable analysis may be explored in future studies has been added to the Discussion.

 6. **Revise tables for clarity.**

**Response:** All tables have been revised for clarity, with clear headings, units, and footnotes to enhance readability and comprehension.

 7. **Improve discussion part about clinical implications.**

**Response:** The Discussion section has been revised and strengthened to better highlight the clinical implications of the study findings.

 8. **Introduction has become too long.**

**Response:** The Introduction has been condensed for improved readability while retaining all essential points and references.

 9. **Use correct tense e.g., “will be” etc.**

**Response:** All relevant sections have been carefully revised for correct tense.

 10. **Page numbers in reference 10 missing.**

**Response:** The reference list has been updated to include the correct page numbers for reference 10.

 We hope that the revisions made have satisfactorily addressed all concerns and have improved the clarity and readability of the manuscript. Thank you very much for your valuable feedback in improving the standard of study and taking the time to address the areas of improvement in our article.

# Reviewer report: Reviewer response for version 1

**DOI:** 10.5256/f1000research.178518.r429880

**Published:** 2025-12-23

**Authors:** Dr Shashidhar A

**Affiliations:** 1 St John's Medical College Hospital, Bengaluru, Karnataka, India

Abstract

 Background should clarify the need for the study. Can delete the rest. Study design to be stated. 

 Give formulae for the indices in methods. Data should be in results. Avoid overconcluding- should be only based on the results. Use Mesh terms

 Main text

 Use STROBE checklist for reporting this observational study throughout

 Summary of evidence is too long should be made crisp and moved to discussion. physiological basis of choosing these indices to be quoted.

 Rationale for sample size should be mentioned. Structure of methods section to be revamped as per author guidelines. How were controls chosen given that the yield of culture may be less. What method of culture was done and when? Was any matching done?

 Consent would be required as the controls are being sampled, data collected and planned for publication

 Study flow is missing

 Unclear when the tests were done and in case of multiple values which one were chosen. Results seem to show an association more than prediction. May need additional analysis like multivariate and logistic regression

Is the work clearly and accurately presented and does it cite the current literature?

Partly

If applicable, is the statistical analysis and its interpretation appropriate?

Yes

Are all the source data underlying the results available to ensure full reproducibility?

Yes

Is the study design appropriate and is the work technically sound?

Yes

Are the conclusions drawn adequately supported by the results?

Partly

Are sufficient details of methods and analysis provided to allow replication by others?

No

Reviewer Expertise:

Neonatology, spesis

I confirm that I have read this submission and believe that I have an appropriate level of expertise to state that I do not consider it to be of an acceptable scientific standard, for reasons outlined above.

# Response

**Published:** 2026-02-12

**Authors:** Smrithi

We sincerely thank Dr. Shashidhar for taking the time to review our manuscript and provide detailed and constructive feedback. We greatly appreciate the suggestions, which have helped us improve the clarity, structure, and scientific rigor of our study. Below, we provide point-by-point responses to each comment.

**Reviewer report:**

 “Abstract - Background should clarify the need for the study. Can delete the rest. Study design to be stated. Give formulae for the indices in methods. Data should be in results. Avoid overconcluding—should be only based on the results. Use MeSH terms.”

**Author response:**

 1. **Background should clarify the need for the study. Can delete the rest.**

 Response: The Abstract has been revised to clarify the study’s rationale, and unnecessary portions have been removed.

**2. Study design to be stated. Give formulae for the indices in methods.**

 Response: The study design has been explicitly stated as a case–control study. The formulae for all inflammatory indices are now included in the Methods.

**3. Data should be in results.**

 Response: Significant study data have been appropriately included in the Results section.

**4. Avoid overconcluding—should be only based on the results.**

 Response: The conclusion has been revised to ensure that statements are strictly supported by study results.

**5. Use MeSH terms.**

 Response: Relevant MeSH terms have been applied to the manuscript keywords.

**Reviewer report:**

 “Main Text - Use STROBE checklist for reporting this observational study throughout. Summary of evidence is too long should be made crisp and moved to discussion. Physiological basis of choosing these indices to be quoted. Rationale for sample size should be mentioned. Structure of methods section to be revamped as per author guidelines. How were controls chosen given that the yield of culture may be less? What method of culture was done and when? Was any matching done? Consent would be required as the controls are being sampled, data collected and planned for publication. Study flow is missing. Unclear when the tests were done and in case of multiple values which one were chosen. Results seem to show an association more than prediction. May need additional analysis like multivariate and logistic regression.”

**Author response:**

**1. Use STROBE checklist for reporting this observational study throughout.**

 Response: We thank the reviewer for this suggestion. The manuscript has been revised to align with the STROBE checklist. We have added a participant flow diagram (STROBE item 13), clarified variables (item 7), and described selection of cases and controls (item 6b). Data collection, timing, and measurement procedures have been clearly described (items 8 and 11). Sample size calculation details have been included (item 10), and Methods section structure has been revised to comply with journal guidelines.

**2. Summary of evidence is too long; should be made crisp and moved to discussion.**

 Response: The summary of evidence has been condensed, with relevant details now included in the Discussion section.

**3. Physiological basis of choosing these indices to be quoted.**

 Response: The physiological rationale for selection of the inflammatory indices has now been incorporated in the Methods section.

**4. Rationale for sample size should be mentioned. Structure of methods section to be revamped as per author guidelines**Response: The Methods section has been revised to provide greater clarity, including the sample size calculation. Sample size was determined using OpenEpi Version 3.01, based on three variables from the reference study by Zhu et al. (2024), with the largest calculated sample size chosen.

**5. How were controls chosen given that the yield of culture may be less?**

 Response: Given the known limitations of blood culture sensitivity in neonatal sepsis, controls were defined based on both microbiological criteria (negative blood culture) and the absence of clinical signs of sepsis ^[33]^. Consecutive eligible preterm neonates meeting these criteria during the study period were included.

**6. What method of culture was done and when?**

 Response: Blood cultures were performed using BACTEC system according to NICU protocol. Samples were collected aseptically after 24 hours of life and processed as per standard microbiological procedures. Culture positivity defined sepsis cases.

**7. Was any matching done?**Response: Formal matching was not performed; however, both cases and controls were drawn from the same source population of preterm neonates admitted to the NICU during the same study period, which helps reduce potential selection bias.

**8. Consent would be required as the controls are being sampled, data collected and planned for publication.**

 Response: As this was a laboratory record–based observational study using routinely collected clinical and laboratory data without any additional sampling or intervention, individual informed consent was waived by the Institutional Ethics Committee of Kasturba Medical College, Mangalore. Hence, we have not included the consent.

**9. Study flow is missing.**

 Response: A study flow diagram has now been included (Figure 1) to illustrate participant selection, allocation into cases and controls, and inclusion in the final analysis.

**10. Unclear when the tests were done and in case of multiple values which one were chosen.**

 Response: Blood samples used for calculation of inflammatory indices were obtained from routine laboratory investigations performed after 24 hours of life. Only values available from routine screening records were included in the analysis.

**11. Results seem to show an association more than prediction. May need additional analysis like multivariate and logistic regression.**

 Response: We thank the reviewer for this insightful comment. We agree that the findings primarily demonstrate associations between inflammatory indices and neonatal sepsis. The study was designed as an exploratory analysis using routinely collected laboratory data, and multivariable logistic regression was not performed due to the limited sample size and absence of comprehensive covariate information. The manuscript has been revised to emphasize associations rather than independent prediction, and ROC analysis is described as assessing diagnostic performance. A statement acknowledging that multivariable analysis may be explored in future studies has also been added to the Discussion.

 We are grateful for the detailed and constructive suggestions. We believe that the revisions have strengthened the manuscript, and we hope that the changes adequately address all concerns and improve the clarity and overall quality of our study.

# Reviewer report: Reviewer response for version 1

**DOI:** 10.5256/f1000research.178518.r426933

**Published:** 2025-12-23

**Authors:** Sanjoy Kumer Dey

**Affiliations:** 1 Bangabandhu Sheikh Mujib Medical University, Dhaka, Dhaka Division, Bangladesh

- Sample size calculation needs to be detailed for better understanding. 
- A flow chart could explain the total number of preterm newborns enrolled (population), cases  (culture positive) and control (culture negative).
- Better to mention where and how investigations were done and who had followed up the babies.
- Data to be made available.

Is the work clearly and accurately presented and does it cite the current literature?

Yes

If applicable, is the statistical analysis and its interpretation appropriate?

I cannot comment. A qualified statistician is required.

Are all the source data underlying the results available to ensure full reproducibility?

No

Is the study design appropriate and is the work technically sound?

Partly

Are the conclusions drawn adequately supported by the results?

Yes

Are sufficient details of methods and analysis provided to allow replication by others?

Partly

Reviewer Expertise:

Neonatal sepsis, IUGR, Neeborn screening, Birth Defect, Online learning

I confirm that I have read this submission and believe that I have an appropriate level of expertise to confirm that it is of an acceptable scientific standard, however I have significant reservations, as outlined above.

# Response

**Published:** 2026-02-12

**Authors:** Smrithi

We would like to express our gratitude to Dr. Sanjoy Kumer Dey for reviewing our article and offering helpful criticism. We are very grateful for the advice, which has improved our study's scientific rigor, clarity and organization. Each comment is addressed below.

**Reviewer Report : **

- Sample size calculation needs to be detailed for better understanding. 
- A flow chart could explain the total number of preterm newborns enrolled (population), cases  (culture positive) and control (culture negative).
- Better to mention where and how investigations were done and who had followed up the babies.
- Data to be made available.

**Author Response :**

**1. Sample size calculation needs to be detailed for better understanding.** 

 Response: We thank the reviewer for this suggestion. The sample size calculation has now been described in detail in the Methods section, including the software used (OpenEpi Version 3.01), the variables considered, and the reference study by Zhu et al. (2024), with the largest calculated sample size chosen for the study.

**2. A flow chart could explain the total number of preterm newborns enrolled (population), cases (culture positive) and controls (culture negative).**

 Response: We appreciate this recommendation. A study flow diagram has now been added (Figure 1) to illustrate the total number of preterm neonates enrolled, exclusions, and the final allocation into cases (culture positive) and controls (culture negative). In addition, a textual description of the participant flow has been included in the Methods section for clarity.

**3. Better to mention where and how investigations were done and who had followed up the babies.**

 Response: Blood cultures were performed using BACTEC system following NICU protocol. Samples were collected aseptically after 24 hours of life and processed according to standard microbiological procedures. Culture positivity defined sepsis cases. As this was a laboratory record–based observational study, no additional clinical follow-up of the neonates was performed; the study utilized only routinely collected laboratory data.

**4. Data to be made available.**

 Response: We have included all the relevant data and the underlying findings under the Data Availability Section.

 We sincerely thank the reviewer once again for their time and valuable suggestions. We believe that the revisions have strengthened the manuscript, and we hope that the changes adequately address all concerns and improve the clarity and overall quality of our study.
